# Prevalence and perceptions of infant massage in India: study from Maharashtra and Madhya Pradesh states

**DOI:** 10.1186/s12887-020-02416-y

**Published:** 2020-11-09

**Authors:** Sarika Chaturvedi, Bharat Randive, Ashish Pathak, Sharad Agarkhedkar, Girish Tillu, Gary L. Darmstadt, Bhushan Patwardhan

**Affiliations:** 1 Dr D Y Patil Vidyapeeth (DPU), Sant Tukaram Nagar, Pimpri, Pune, 411018 India; 2 Centre for Clinical Global Health Education, Johns Hopkins University, Baltimore, MD USA; 3 Department of Paediatrics, R D Gardi Medical College, Ujjain, MP India; 4 Global Health (IHCAR) / Dept of Public Health Sciences, Karolinska Institutet, 171 77 Stockholm, Sweden; 5 Department of Pediatrics, Dr D Y Patil Medical College Hospital and Research Centre, Pimpri, Pune, 411018 India; 6 Centre for Complementary and Integrative Health, Interdisciplinary School of Health Sciences, Savitribai Phule Pune University, Pune, 411007 India; 7 Department of Pediatrics, Stanford University School of Medicine, Stanford, CA USA

**Keywords:** Oil massage, Emollient therapy, Traditional practices, Infant care, Ayurveda

## Abstract

**Background:**

Knowledge of the prevailing infant care practices and their effects is important to inform practice and public programs. Infant massage is a traditional practice in India but remains less studied. This study was conducted to study the prevalence and perceptions of infant massage practices in two states of India.

**Methods:**

A total of 1497 caretakers of children under 18 months of age were interviewed in a cross-sectional study at immunisation units of medical schools in Maharashtra (MH) and Madhya Pradesh (MP) states and through home visits in villages in MH during March through August 2018.

**Results:**

Infant massage was a prevalent practice (93.8% [95%CI: 92.4,94.9]) in both study states – 97.9%[95%CI:96.9,98.8] in MH and 85.3%[95%CI: 81.9,88.3] in MP – and the prevalence did not vary between male (94.5%) and female (93.5%) infants (*p* = 0.44). Massage was mostly initiated in the first week of life (82%); it is widely viewed as a traditional practice. It was common to massage the baby once daily (77%), before bathing (77%), and after feeding (57%). Massage was mostly conducted using oils (97%). In MH, preferred oils were a sesame oil-based proprietary traditional medicine oil (36%) and coconut oil (18%) while olive (29%) and mustard (20%) oils were most popular in MP. Commonly reported application techniques included gentle massage with minimal pressure, pressing (30%) and manually stretching certain joints (60%). Commonly reported perceived benefits of infant massage included increased bone strength, better sleep and growth, while no harm was perceived (95%).

**Conclusion:**

Infant oil massage is a highly prevalent traditional practice in MH and MP. Clear guidance on the use of massage, choice of oil, and techniques for application is required to optimize benefits and minimize risks of this popular traditional practice.

**Supplementary Information:**

The online version contains supplementary material available at 10.1186/s12887-020-02416-y.

## Background

Newborn, infant and child health is a high priority area for sustainable development. Mortality indicators are improving globally. Neonatal mortality is on the decline with the world’s neonatal mortality rate falling from 37 deaths per 1000 live births in 1990 to 18 per 1000 live births in 2018 [1]. The annual rate of reduction in global under-five mortality increased from 2% in 1990–2000 to 3.8% in 2000–2018 [1]. Despite this global progress, an estimated 5.4 million children under age 5 died in 2017, leaving much to be achieved in low- and middle-income countries (LMICs) where approximately 98% of global under-five deaths occur. Infant mortality in these regions is about 17 times higher than in developed regions [1]. Inequalities prevail within regions and between socioeconomic classes in the regions [2].

Global efforts to improve newborn and child health outcomes include interventions across the continuum of care such as prenatal care, skilled care at birth, immediate newborn care, and post-partum maternal and newborn care. Social determinants of health such as socio-economic status, education, gender, caste, race, disability, age and access to quality medical care influence infant/child health outcomes and are important underlying considerations for lasting improvements. Literature suggests the need to study and adapt interventions that are culturally acceptable, including community-based interventions [3, 4]. Infant care practices tend to be region- and culture-specific and influence child health outcomes. Cultural practices are sometimes beneficial and sometimes harmful, and for some there exists no clear evidence of benefit or harm. Knowledge of the prevailing child care practices and their effects is hence important to inform practice and public programs [5].

For centuries, families in India and many parts of Asia have been massaging the newborn and the infant, generally with an oily substance. Instilling oily substances in the infants’ ears and nose and applying an oil-based collyrium in the eyes are also popular practices in India [6]. Traditions are likely to undergo modifications with access to information and diverse exposures. For instance, child birth that was perceived more as a natural process conducted at home with support from traditional birth attendants has shifted to the medical domain in the control of skilled birth attendants globally. The proportion of home births and births by traditional birth attendants in India has declined from 74% in early 1990s to about 22% in 2015–16 [7, 8]. The shift in place of delivery and related care is likely to have influenced child care practices. A study of the epidemiology and perceptions of these practices in current times can provide the basis for region-specific appropriate guidance for infant care.

Massage in babies, with and without oils, has been researched and several benefits have been reported. These include improved anthropometric parameters such as weight gain velocity and length [9]. A meta-analysis of infant oil massage found it to be effective at promoting physical growth and had limited risk of adverse skin reactions [10]. A majority of reports on effects of topical applications to infant skin, termed as ‘emollient therapy,’ are from hospitalised babies that are born premature and require intensive care [11]. Literature on massage of well babies in community settings is scarce. Also, less is known of the current prevalence of massage in different parts of India. Further details of the practice in India such as what are the commonly used substances for massage, how often and for how long is massage given, and the perceived benefits or harms of massage have not been reported. The objective of this paper is to address this knowledge gap based on a study in two parts of India, namely Maharashtra (MH) and Madhya Pradesh (MP).

## Methods

### Settings

This study was conducted in Maharashtra (MH) state in Western India and Madhya Pradesh (MP) in central India. These two regions were chosen purposively considering authors’ access to potential study sites. MH is a relatively developed state with better health indicators than MP (Supplementary file 1).

### Design

This was a cross sectional study.

### Data collection

Data were collected at immunisation units of private medical college hospitals (*n* = 1 in Pune, MH and *n* = 1 in Ujjain, MP). These private academic institutes offer services ranging from primary care to super speciality and are popular in the study locations. In MP, data were also collected at the immunisation unit of the district hospital (*n* = 1) and a charitable trust hospital (*n* = 1). The district hospital is a public facility providing highly subsidised services for the poor and at small costs to others. The charitable hospital is a reputed not for profit private institute offering speciality services at relatively lower charges than private setups. All chosen hospitals are large multi-speciality hospitals with high patient turn around. The hospitals were chosen purposively in view of feasibility. In MH, apart from the medical college hospital, data were collected through a community-based survey and home visits in 26 villages in Purandar taluka in Pune district. The choice of villages was purposive based on accessibility to data collectors. Data collection took place during March through August 2018.

A structured questionnaire was developed based on the clues from available literature and authors’ knowledge of traditional child care practices. (Supplementary file 2) The questionnaire had 38 items in total seeking details such as if they massage the infant, who performs the massage, how often, how long, what do they use for massage and the reasons thereof. A few open-ended questions were included to assess perceptions such as potential harms and benefits of infant massage and precautions taken during infant massage. The questionnaire was translated in the local languages: Marathi for the MH site and Hindi for the MP site. The questionnaire was pilot tested in both study areas. Most data collectors were females - four in MH and four in MP - and had prior experience in conducting surveys. They received 2 days training in administering the questionnaire. The data collectors were supported and supervised on-site by team members who were either medical doctors or experienced social workers.

At the immunisation units of selected medical schools, the data collectors approached care-takers of babies up to 18 months of age who were brought for their scheduled immunisation. This age criterion was chosen as immunisations advised in this age range are more frequent and to avoid recall bias. These babies were not sick. The questionnaire was administered as the care-takers waited for their turn. In rural MH, data collection was by trained community health workers through home visits in the selected villages. The data collectors explained the study to the potential participants and obtained informed consent.

### Sample size

For the prevalence objective, the desired sample size to detect a 50% prevalence of infant massage with a precision of +/− 5% was 384. In order to obtain variation and details of the practices under study, we planned to include 500 respondents each from the study hospitals in MH and MP and 500 respondents from the villages in MH; thus, the total intended sample size was 1500 respondents.

The study protocol was approved by the Institutional Ethics Committee of Savitribai Phule Pune University in MH and the R D Gardi Medical College in MP. Consent to participate in this study was provided by the care takers interviewed.

Descriptive analyses were conducted. Data were analysed using statistical software STATA v 11.0. Chi square test was used for comparing categorical variables. A *p*-value below 0.05 was considered significant. Confidence intervals were determined at 95%.

## Results

### Characteristics of the study sample

The background characteristics of the babies enrolled in the study (*n* = 1497) are described in Table 1. Nearly half (45%) of the included babies were under 6 months of age. Most of the babies were born at full term (> 37 weeks) (86%) and had normal birth weight (81%), while 1%were very low birth weight (< 1500 g) and 17% were low birth weight (< 2500 g). Most of the births occurred at facilities located at district-level or more peripherally and most were in private health facilities or medical college hospitals (55%) compared to public sector facilities. Nearly two thirds of the babies (64%) were born by vaginal delivery; the caesarean section rate was 35%. Eleven percent were born preterm and 13% required hospitalisation soon after birth (median 5 days after delivery, IQR 3–9 days).

**Table 1:** Characteristics of study sample (*n* = 1497)

Characteristic	Description	Number (%)
Age	Up to 3 months	347 (23.29)
	3–6 months	325 (21.81)
	6-12 months	589 (39.53)
	12–18 months	229 (15.37)
Sex	Male	848 (57.26)
	Female	633 (42.74)
Birth weight	Below1500 grams	16 (1.08)
	1500–2499 g	263 (17.69)
	> 2500 g	1208 (81.24)
Mode of delivery	Vaginal	967 (64.73)
	Caesarean	527 (35.27)
Gestational age at birth	Preterm	165 (11.07)
	Term	1292 (86.71)
	Post-dated	33 (2.21)
Birth facility location	District	648 (43.67)
	Town	707 (47.64)
	Village	129 (8.7)
Birth facility type	Private facility/hospital	510 (34.13)
	Private medical college hospital	308 (20.62)
	Public facility/hospital	533 (35.68)
	Public medical college hospital	143 (9.57)

### Infant massage practices

The practice of massaging the baby at least once a day was highly prevalent (93.8% [95%CI: 92.4,94.9].). The prevalence was higher in MH (97.9% [95%CI: 96.9,98.8]) than MP (85.3% [95%CI:81.9,88.3]) (Table 2). Seventy-seven percent of the respondents were giving massage to their babies at the time of this survey. Of those who had discontinued giving massage, most had continued it until the baby was about 10 months old.

**Table 2:** Comparison of massage practices between study states

Prevalence of massage practice		Total *n* = 1497(%)	MH *n* = 1000 (%)	MP *n* = 497 (%)	*X*^2^ Statistic	*P* value^#^
Received massage (overall)	Yes	1403 (93.8)	979 (98.0)	424 (85.3)	91.61	< 0.01^*^
	No	93 (6.2)	20 (2.0)	73 (14.7)		
Receiving massage at 3 months	Yes	308 (89)	231 (95.1)	77 (74.8)	30.50	< 0.01^*^
	No	38 (11)	12 (4.9)	26 (25.2)		
Receiving massage at 6 months	Yes	293 (90.1)	165 (93.2)	128 (86.5)	4.11	0.04^*^
	No	32 (9.9)	12 (6.8)	20 (13.5)		
Receiving massage at 12 months	Yes	453 (76.9)	293 (72.5)	160 (86.5)	13.92	< 0.01^*^
	No	136 (23.1)	111 (27.5)	25 (13.5)		
Receiving massage at 18 months	Yes	97 (42.4)	57 (32.6)	40 (74.1)	29.11	< 0.01^*^
	No	132 (57.64)	118 (67.4)	14 (25.9)		

*MH* Maharashtra, *MP* Madhya Pradesh

*significant at *p* < 0.05 ^#^ Comparisons using Chi-squared test

Overall, the prevalence of massage declined with age of the child from 89% receiving massage at 3 months to 42% at 18 months. However, in MP the prevalence of about 75% at age 3 months was sustained at 18 months, unlike in MH where it declined from 95% at 3 months to 33% at 18 months (Table 2).

There was no difference in the overall prevalence of massage by sex of the infant (94.5% in males vs 93.5% in females, *p* = 0.44). Also, there was no difference in massage prevalence by sex in either state (MH: males 97.6.% vs females 98.3% *p* = 0.41 and MP males 86.2% vs females 85.1% *p* = 0.74) (Table 3).

**Table 3:** Massage practices by infant sex^a^

Total/State	Infant massage	Male *n* = 633 (%)	Female *n* = 848 (%)	*X*^2^ Statistic	*P*-value
Total	Yes	598 (94.5)	793 (93.5)	0.58	0.44
	No	35 (5.5)	55 (6.5)		
MH	Yes	448 (97.6)	530 (98.3)	0.66	0.41
	No	11 (2.4)	9 (1.7)		
MP	Yes	150 (86.2)	263 (85.1)	0.10	0.74
	No	24 (13.8)	46 (14.9)		

*MH* Maharashtra, *MP* Madhya Pradesh

^a^Comparisons using Chi-squared test

Massage was mostly initiated in the first week of life except in case of sick babies. The majority of mothers had initiated massage as a traditional practice (82%) and it was rarely initiated on the advice from a healthcare provider (4%). It was common to massage the baby once daily (77%); about one fifth of the respondents, mostly from MP, reported massaging twice daily (19%). The average duration of a massage session was 15 min (IQR:10–20). Massage to the baby was mostly given by the elderly women in the family, generally grandmothers (39%) while a few had the services from a traditional birth attendant (*dai*) (10%) with a median expense of INR 1000 per month. Massage was mostly performed before bathing (77%), after feeding (57%) and before sleep (45%).

Oil was the preferred substance used to massage the baby (97%). Rarely other substances were used including butter, clarified butter, body lotion or a mix of gram flour and turmeric powder. A variety of oils were used for baby massage (Table 4) with much variation by state. In MH a proprietary Ayurvedic oil (sesame oil treated with herbs) was most commonly used (36%) followed by coconut oil (18%), while in MP the preferred oils were olive (29%) and mustard (20%). Exceptional to this variety of oils, a non-traditional proprietary product (mineral oil preparation marketed as baby oil) was used by 15% in MH and 8% in MP.

**Table 4:** Types of oil used for infant body massage

Oil used for massage	Maharashtra	Madhya Pradesh	Total
Proprietary Ayurvedic^a^ oil (sesame oil base)	36.2	18.3	30.8
Coconut oil	17.6	2.9	13.2
Proprietary baby oil^b^ (mineral oil base)	15.5	8.4	13.4
Mustard oil	5.9	19.5	10.0
Olive oil	0.4	29.4	9.0
Cooking oil^c^	7.9	0	5.5
Proprietary oil- (olive oil base)	8.5	5.5	7.6
Other oil	4.9	13.2	7.3

^a^Ayurvedic- Based on Traditional Indian medicine system, mostly sesame oil treated with herbs mentioned in the Ayurvedic texts

^b^Petroleum-based mineral oil with fragrance marketed as baby oil

^c^Edible plant seed oil sunflower, safflower and/or soybean oil, rarely groundnut oil

The choice of oil for massage was informed by advice from family/friends (37%), traditional know-how (23%) or personal experience (27%); it was less often on seeking advice from a trained provider (11%). Only 1% of mothers reported that their choice of oil was influenced by media advertisements.

Almost all mothers reported using lukewarm oil for massage. It was a common practice to add garlic or fenugreek seeds to the oil when heating it while a few reported adding carom seeds, nutmeg powder or turmeric powder to the oil.

#### Massage techniques

All mothers described the process of applying oil as a gentle massage with minimal pressure. Thirty percent reported pressing body parts during massage, commonly the joints of the hands and legs, the umbilicus and the lateral sides of the nose. Mothers also reported manually stretching certain joints at the time of massage (60%); these were commonly the large joints of the extremities such as elbow and knee. Gently pressing on the sides of the nose bridge with upward lifting of the skin was also often reported.

The most common reason for not massaging babies was unavailability of a person skilled to provide baby oil massage. Other frequently cited reasons included having had or heard of others’ negative experiences, advice from a health care provider discouraging massage, and lack of time.

#### Perceptions about infant massage

Baby massage was believed to be a useful practice. Commonly reported perceived benefits included increased bone strength, better sleep and growth. Massage was said to aid in early walking and sitting by the baby. Many mothers perceived that with massage, babies are less irritable and fussy while some suggested that babies who received massage smiled more often. Massage was perceived to make the baby active and playful. Some thought that massage is relaxing and refreshing to the baby while a few emphasised that it is like an exercise to the baby. A few reported that massage promotes weight gain and is good for the skin. More rarely respondents mentioned that babies receiving massage are sick less often.

It was a common perception that massage could not be harmful (95%); upon probing, the few instances of adverse events were thought to be due to negligence or an accident such as applying overheated oil or improper technique like forceful massage or exposure to cold. A few mothers cautioned against erroneous choice of oil for massage: specifically, an oil perceived to be ‘cold,’ such as coconut oil was thought to be a bad choice in cold weather, and similarly a ‘hot oil’ like mustard oil was thought to be a poor choice in the summer. Allergy to the massage oil manifested as skin rash was reported by one respondent.

#### Provider advise about massage and other practices

Mothers indicated that the practice of infant oil massage was not discussed (83%) with a health care provider nor did any health care provider routinely advise about it. Mothers of sick babies were generally advised to not massage the baby except one reported that she received advice to frequently massage a low birth weight baby for better weight gain. In the few reports where there was a discussion about baby massage, the doctors commonly suggested proprietary, non-traditional baby oil and less commonly proprietary Ayurvedic oil; coconut oil was also suggested occasionally.

## Discussion

Knowledge of prevalent child care practices is important to appropriately tailor public health programs. This report on the traditional child care practice of oil massage in two states of India brings forth several important details of the practices, hitherto unreported in the Indian context.

Newborn infants have a relatively thin epidermis compared to adults, making it more permeable to loss of water and heat, and entry of pathogens and toxins [12, 13]. Also, given the high body surface to body weight ratio in infants, applications to infant skin can have more effects than in adults and are an important consideration for infant health. This report of prevailing infant care practices in community settings is hence relevant to inform infant health measures.

A high prevalence of the traditional practice of infant massage was found in MH and MP states of India despite ‘modernisation’ of the society. Although we did not find any past reports of prevalence of infant massage from the study states, the report of high prevalence of the practice of oil massage in a relatively older study from southern India [14] was echoed (97%) in a subsequent study about four decades later from another South Indian state [6]. This continued practice despite provider recommendation indicates peoples’ strong belief in the traditional practice and perceived benefits of infant massage. Although MP is a relatively less developed state, the prevalence of infant massage was lower than in MH, contradicting the notion that traditions weaken with the current models of development and suggesting that factors unrelated to development may influence the practice.

Community-based studies on neonatal care practices report high prevalence of massage in South Asian countries. Near universal massage of newborns is reported from Nepal [15–18], Pakistan [19, 20] and Bangladesh [21, 22]. A recent report finds massage to also be normative in four African countries [23] with regional and tribe-based variations in emollients used for massage while a previous report also found the practice to be variable, although not universal, in the African region [24].

Despite the high prevalence of massage in this study, it was hardly discussed with a health provider. This may be indicative of a disconnect of the practitioners with the patients’ lives or possibly a ‘normalisation’ of the practice - the providers may be well aware of the practice as routine at homes. The perceptions of providers need to be studied. The fact that there were no differences in the practice for male or female infants is noteworthy given the son preference in the study area, especially in MP [25, 26]. We found that mostly women in the family performed the infant massage. This obviates the need of a trained person and offers added advantages of emotional bonding with the infant [27].

Plant-based oils were the preferred substance for massage, similar to previous reports from Asian countries. Topical oils as used in infant massage can serve a nutritional purpose as sources of essential fatty acids, especially for preterm and low birth weight infants [28]. Solanki et al. also reported topically applied oil raised fatty acid levels in infants and may have nutritional value [29].

Topically applied oils act as a barrier to skin disruption and provide lipids that enhance the skin barrier function. Weak skin barrier function results in susceptibility of infants to infections, often leading to bloodstream infections, need of broad spectrum antibiotics with known risks, and possibly fatalities. Skin barrier function is known to be weak in preterm infants, but it is important to note that the skin of even term infants in developing country contexts may be suboptimal due to intrauterine malnutrition [30]. The choice of oil for infant massage is important as different oils are known to have varying effects. Topical applications containing a physiologic balance of epidermal lipids (3:1:1:1 M ratio of cholesterol,ceramide,palmitate, and linoleate) are known to be optimal for skin barrier repair [31, 32]. In MH, sesame oil-based proprietary Ayurvedic oil was mostly used followed by coconut oil. Sesame oil massage was found to have beneficial effect on infant growth and sleep and was better than mineral oil [33]. The antibacterial, antifungal and nutritional effects of sesame oil are also proven by laboratory studies [34–36]. However, further research is needed on impacts of sesame oil on measures of infant health. Coconut oil also has antimicrobial activity against many pathogens as it contains monolaurin, a short-chain fatty acid [37], however, it can also raise skin pH which can disrupt barrier function. Topical coconut oil application on preterm infant skin showed reduced trans-epidermal water loss (TEWL) and better skin condition than standard care [38] while coconut oil massage showed improved anthropometric parameters compared to mineral oil massage [39]. A randomised trial in preterm infants in Pakistan found improved skin condition and weight and reduced blood stream infections with coconut oil application [40]. Studies show mustard oil to be harmful to infant skin, and olive oil increased TEWL as a measure of skin barrier function in a mouse model of infant skin [41]. Detrimental effect of topical olive oil on adult skin integrity has also been documented, discouraging use of olive oil for infant massage [42]. In MP, mustard oil was most commonly used followed by olive oil. Our findings should alert healthcare providers to discuss infant massage and advise against the use of these neat oils. Studies from Nepal [15–18] and Bangladesh [22] also report the high prevalence of mustard oil use for newborn massage. Traditionally made choices may find reasoning in traditional medicine. However, the Indian traditional medicine Ayurveda also discourages use of mustard oil and supports use of sesame and coconut oil for infant massage. Our findings of perceptions about ‘hot’ and ‘cold’ oil, and the altered choices according to seasonality and the processing of oil for massage by heating it with carom or fenugreek seeds relates to the Ayurvedic science that supports this processing to make the oil more suitable for the infant body. Notably, Agarwal et al. documented a better effect of plain sesame oil than sesame oil treated with certain herbs on infant growth, indicating the use of herbs and processing of the oil by heating may alter the properties [33]. Appropriate use of the knowledge base from Ayurveda for choosing the herbs and processing the oil is hence important. Moreover, additional research is needed on the impact of variations of oils on infant health. Notably, evidence for oils other than the above-mentioned oils found popular in our study favours sunflower seed oil for infant massage especially in preterm and low birth weight infants [21, 43, 44].

Our findings of techniques of massage including gentle massage with stretching of extremities and slight pressure at large joints are not only novel but interesting for the aspect that these techniques are uniformly reported across the sample. The technique of massage used by Mathai et al. – two phases of tactile stimulation, one in supine and another in prone position, followed by a final phase of kinesthetic stimulation [45] – is similar to the practices reported by mothers in our study. A review of literature suggests that moderate pressure is essential for massage effects on infant growth and development [46]. However, vigorous massage can be harmful to the skin barrier, especially in preterm and malnourished infants with a thin, vulnerable skin barrier. We therefore recommend standardisation of the techniques of infant massage and to routinely provide appropriate demonstration and instruction to the caretakers. Given the rising proportion of facility births in LMICs, postnatal health provider contact provides an important opportunity for this education.

Besides the effect of the oil used for infant massage, the human touch during massage is an important aspect. A trial found sensoritonico motor touch and application of vegetable oils to healthy preterm infants improved weight gain and neurological development [47]. Therapeutic touch is gaining increased interest especially for preterm infants [48]. Tactile-kinesthetic stimulation in the form of massage administered to well, preterm infants was found to have a beneficial effect on growth and behavioural development and no adverse effects on physiologic parameters [45].

Perceived benefits of massage include healthy growth and development; improved sleep, bone strength, and weight gain; and relief of stress and promotion of relaxation of the infant. Further research should explore these parameters in community settings as also recommended by the Cochrane review by Bennett et al. [49].

### Limitations

Our findings are from a cross sectional study conducted at immunisation visits and partly at homes. It is possible that those who do not visit immunisation clinics may follow different practices than we report. However, as immunisation coverage is high in the study area, we do not believe it would affect our findings. In addition to sampling at immunisation clinics, in MH we also conducted home surveys to increase representativeness of rural areas. This slight difference in sampling between states is unlikely to affect results for this cross-sectional study. We have included infants until 18 months of age and while practices are highly prevalent, chances that there is failure to recall are small but cannot be denied. Our findings may not be widely applicable to the entire country, as the proportion of low birth weight infants in our sample is low, and thus may not include a representative range of vulnerable infants.

## Conclusions

Infant oil massage is a highly prevalent traditional practice in MH and MP states of India. Overall, our findings suggest the need for clear guidance on safe and effective uses of traditional skin care practices, especially in contemporary times when the preparations may differ from in the past. Given the magnitude of the practice in India, further research to optimise the benefits and reduce potential risks of infant oil massage is required.

## Supplementary Information

**Additional file 1.** Supplementary File 1: Selected health and development indicators of the study states.

**Additional file 2.** Study questionnaire.

## Data Availability

The datasets used and/or analysed during the current study are available from the corresponding author on reasonable request.

## Abbreviations

IQR: Inter quartile range
LMIC: Low and middle income countries
MH: Maharashtra
MP: Madhya Pradesh
TEWL: Trans epidermal water loss
