# Supplementary material for: Prevalence and perceptions of infant massage in India: study from Maharashtra and Madhya Pradesh states
Source: BMC Pediatr. 2020 Nov 9;20:512. doi: 10.1186/s12887-020-02416-y (PMC7650175; doi:10.1186/s12887-020-02416-y)
Supplement: Supplementary file 1 — Additional file 1. Supplementary File 1: Selected health and development indicators of the study states. [file 12887_2020_2416_MOESM1_ESM.docx]

Supplementary File 1: Selected health and development indicators of the study states

| Indicator | Maharashtra | Madhya Pradesh | India |
| --- | --- | --- | --- |
| Poor population (%) * | 17 | 32 | 22 |
| Total Fertility Rate^ | 1.9 | 2.3 | 2.2 |
| Female Literacy (%) ^ | 80 | 60 | 68 |
| Institutional births (%) ^ | 90 | 81 | 79 |
| Maternal mortality ratio/100000 births^@^ | 68 | 221 | 167 |
| Infant mortality rate/1000 births^ | 24 | 51 | 41 |
| Underfive children underweight (%) ^ | 36 | 43 | 36 |

*Reserve Bank of India 2013

^National Family Health Survey 2015-16

@Sample Registration Survey 2011-13
