# Supplementary material for: Prevalence and perceptions of infant massage in India: study from Maharashtra and Madhya Pradesh states
Source: BMC Pediatr. 2020 Nov 9;20:512. doi: 10.1186/s12887-020-02416-y (PMC7650175; doi:10.1186/s12887-020-02416-y)
Supplement: Supplementary file 2 — Additional file 2. Study questionnaire. [file 12887_2020_2416_MOESM2_ESM.pdf]

Centre for Complementary and Integrative health
Savitiribai Phule Pune University

Survey on child care practices

1. ID: ☐☐☐☐☐☐
2. Age in months: ☐☐
3. Number of live babies born to the mother: ☐
4. Recent Baby's Date of birth: ☐☐☐☐☐☐
5. Gender: 1. ☐ Female 2. ☐ Male
6. Mode of delivery: 1. ☐ Vaginal 2. ☐ Abdominal
7. Location/Place of delivery: 1. ☐ Large city 2. ☐ Town 3. ☐ Village
8. Place of delivery: 1. ☐ Private hospital, small 2. ☐ Private hospital, large/corporate
   3. ☐ Private Medical College 4. ☐ Government/corporation hospital
9. Duration of pregnancy: 1. ☐ Completed full term 2. ☐ ………weeks before full term
   3. ☐ ……….weeks after full term
10. Birth weight: ☐ Kgs
11. Any problems to baby at birth……………………………………………………………………….
    ………
12. Hospitalisation to baby after birth: 1. ☐ Not hospitalised
    2. ☐ Hospitalised for …….. days
13. Do you routinely put anything in baby's ear: 1. ☐ No 2. ☐ Yes
    What if yes: ……………………………
14. Do you routinely put anything in baby's nose: 1. ☐ No 2. ☐ Yes
    What if yes:………………………………
15. Do you routinely put anything in baby's eyes: 1. ☐ No 2. ☐ Yes
    What if yes:…………………………
16. Do you routinely put anything on baby's fontanalle: 1. ☐ No 2. ☐ Yes
    What if yes:……………….

17. What else do you apply to the baby: 1. ☐ Powder 2. ☐ Cream 3. ☐ Shampoo
4. ☐ Other than above, What if so……………………..
5. ☐ Do not apply anything

18. Do you give massage to the baby: 1. ☐ Yes, currently giving ;
Plan to continue giving massage till………months/years
2. ☐ Discontinued after……..
3. ☐ No, did not give

If NO, skip to Question 34.

19. When after birth of the baby did you start giving massage? ☐ days

20. Why did you start giving massage to the baby?
1. ☐ Traditional practice 2. ☐ Told by family elders/friends
3. ☐ Told by doctor/nurse 4. ☐ Told by dai/traditional care giver
5. ☐ Other than above Specify here………………………………………………………………..

21. Who gives the massage to your baby generally?
1. Parents: ☐ Mother ☐ Father
2. Relative: ☐ Elderly ☐ Not elderly Relation to baby:……………
3. Neighbour/acquaintance: ☐ Female ☐ Male
4. Dai: ☐ Female ☐ Male
5. Hired professional: ☐ Profession- ………………
6. Other than above: ☐ Specify: ……………………………

22. How often is massage given to your baby? …………….times/ day or ………times / week

23. How long does a massage session last? …………minutes

24. When do you generally give massage- any relation to bath, feeding or sleep?

**Bath**	**Feeding**	**Sleep**
☐ Before bath	☐ Before feeding	☐ Before Sleep
☐ After bath	☐ After feeding	☐ After waking up
☐ Not related to bath	☐ Not related to feeding	☐ Not related to sleep time

☐ Specify any other time followed if any:…………………………………………………………………………..

25. What substance do you use mostly for massage?
1. ☐ Oil : Specify ….:…….. 2. ☐ Ghee : Specify……………
3. ☐ Butter: Specify:……… 4. ☐ Powder: Specify:……………..
5. ☐ Lotion/cream : Specify:……… 6. ☐ Other than above: Specify: …………….

26. What are the reasons for this choice (of substance for massage)?
   1. ☐ Own experience
   2 ☐ Recommended by family elders/friends
   3. ☐ Recommended by doctor/nurse
   4. ☐ Recommended by pharmacist
   5. ☐ By Traditional knowhow
   6. ☐ Other than above……………………………

27. Do you make any preparation for the massage substance (such as heating oil etc.)?
   …….

28. Where do you get the massage substance from:
   1. ☐ Make at home
   2. ☐ Buy from market

29. How much does the massage substance you use cost? Rs ………………/month

30. Do you press at any specific areas of the baby's body during massage?
   1. ☐ No 2. ☐ Yes If yes, which points: …………………………………………..

31. Do you stretch any body parts of the baby along with massage?
   1. ☐ No 2. ☐ Yes if yes, pl describe:……………………………………………….

32. What precautions do you take while giving massage?
   1…………………………………………………………………………………………
   2. ………………………………………………………………………………………
   3……………………………………………………………………………………….
   4. ……………………………………………………………………………………….

33. Are there any conditions when you avoid giving massage?
   1……………………………………………………………………………………………..
   2…………………………………………………………………………………………….

34. Why did you not give massage to your baby?
   1. ☐ Advised so by doctor/nurse
   2. ☐ Advised so by family elders/friends
   3. ☐ Own experience
   4. ☐ Lack of time
   5. ☐ No skilled person available
   6. ☐ Other than above:Specify here……….. …

35. What do you think are the benefits of massage, if any?
   1…….
   2……
   3…….
   4……

36. What do you think could be the harm from massage, if any?
   1………
   2……
   3…….
   4. ……..

37. Did you consult a doctor about any of the above baby care practices:

1. Massage A. ☐ No B . ☐ Yes If yes, mention advise: ……………………………

2. Instilling in fontanalle A. ☐ No B. ☐ Yes If yes, mention advise: …………………

3. Instilling in eye A. ☐ No B. ☐ Yes If yes, mention advise: ………………………

4. Instilling in ear A. ☐ No B. ☐ Yes If yes, mention advise: ……………………

5. Instilling in nose A. ☐ No B. ☐ Yes If yes, mention advise: ………………..

Name and address of facility where form is filled:
……….

Date of form filling:………………………………….

Filled by: Name and Sign…………………………………………………

Checked by: Name and Sign …………………………………..

Supervisor: Name and sign …………………………………..

Data entered by: …………….
